# Serum MMP-2 as a potential predictive marker for papillary thyroid carcinoma

**DOI:** 10.1371/journal.pone.0198896

**Published:** 2018-06-27

**Authors:** Yunpeng Shi, Chang Su, Haixia Hu, He Yan, Wei Li, Guohui Chen, Dahai Xu, Xiaohong Du, Ping Zhang

**Affiliations:** 1 Department of Hepatobiliary and Pancreatic Surgery, The First Hospital of Jilin University, Changchun, Jilin, China; 2 Department of Thyroid Surgery, The First Hospital of Jilin University, Changchun, Jilin, China; 3 Department of Emergency, The First Hospital of Jilin University, Changchun, Jilin, China; 4 Department of Pathology, Jilin City People’s Hospital, Jilin, Jilin, China; University of South Alabama Mitchell Cancer Institute, UNITED STATES

## Abstract

**Objective:**

The prevalence of papillary thyroid carcinoma (PTC) is rising rapidly. However, there are no reliable serum biomarkers for PTC. This study aimed to investigate the validity of preoperative serum matrix metalloproteinase-2 (MMP-2) as a biomarker for predicting prognosis of PTC after total or partial thyroidectomy.

**Methods:**

Male patients with PTC or a benign thyroid nodule (BTN) and healthy controls (HCs) were retrospectively included. Receiver operating characteristic (ROC) curves were constructed to evaluate the performance of preoperative serum MMP-2 in diagnosing PTC, predicting lymph node metastasis (LNM), and predicting structurally persistent/recurrent disease (SPRD). Multivariate logistic regression and Cox regression were applied to identify independent risk factors for SPRD.

**Results:**

The preoperative serum MMP-2 concentration in the PTC group was higher than those in BTN and HC groups. The concentration of postoperative serum MMP-2 decreased in comparison with pre-operation. ROC curves showed that serum MMP-2 could differentially diagnose PTC from BTN at the cutoff value of 86.30 ng/ml with an area under the curve (AUC) of 0.905 and could predict central LNM (CLNM) at the cutoff value of 101.55 ng/ml with an AUC of 0.711. Serum MMP-2 ≥101.55 ng/ml, age ≥45 years, and advanced TNM stage were independent risk factors for CLNM. Patients with SPRD had a higher median MMP-2 level (149.22 ng/ml) than patients without SPRD (104.55 ng/ml). Serum MMP-2 at the cutoff value of 144.04 ng/ml could predict SPRD in PTC patients with an AUC of 0.803. Advanced TNM stage and serum MMP-2 ≥144.04 ng/ml were independent risk factors for SPRD. Patients with serum MMP-2 ≥144.04 ng/ml had a worse clinical outcome than those with MMP-2 <144.04 ng/ml.

**Conclusion:**

Preoperative serum MMP-2 may serve as a biomarker for diagnosing PTC and a predictive indicator for LNM and SPRD in male patients with PTC.

## Introduction

Papillary thyroid carcinoma (PTC) is the predominant histological type of thyroid malignancy, accounting for 80%‒85% of cases [1,2]. Previous studies always focused on female patients with PTC. However, male patients with PTC are common. Although differentiated PTC carries a favorable prognosis, it causes a postoperative recurrence rate as high as 30% during long-term follow-up [3]. A few biomarkers have been identified for the diagnosis of PTC, including EHD2 [4], Notch signaling [5], HER-2/neu [6], and CXCL12 [7], without clear evidence of their efficacy for clinical use. Therefore, the identification and validation of reliable biomarkers for diagnostic and prognostic prediction of PTC is urgently needed in clinical work.

Matrix metalloproteinases (MMPs) are zinc-dependent protein and peptide hydrolases. MMP-2 is a member of the MMP family, and it functions in diverse biological processes through the degradation of extracellular matrix. MMP-2 has been implicated in carcinogenesis and invasive features of various types of cancers [8–12]. In feline mammary carcinoma, the expression of MMP-2 in peripheral neoplastic foci of tumors with high histological grade (grade III) was higher than that in low-grade tumors[13]. Overexpression of MMP-2 was found in prostate cancer tissue, as compared with benign prostatic hyperplasia tissue, and the level of MMP-2 expression was correlated with clinical stage [8]. A study among the Tunisian population reported that a higher intensity of MMP-2 staining may be associated with colorectal cancer progression and invasion [14]. Serum MMP-2 has also been considered as a useful tool for differentiation of clinical stages of tumors and prediction of cellular differentiation grade in endometrial cancer [15]. Patients with elevated serum MMP-2 concentration were found to be at greater risk of central nervous system metastasis [16]. On the contrary, detection of the serum MMP-2 level has yielded conflicting results in various populations. A previous study demonstrated that a higher serum level of MMP-2 was correlated with favorable outcome in inflammatory breast cancer treated with bevacizumab-based chemotherapy [17]. Still, sequential detection of serum MMP-2 has been suggested as a strategy for assessing the chemotherapy response in optimally operated patients with ovarian cancer[18].

Some studies have already reported the significance of MMP-2 in the diagnosis and prognosis of PTC. Immunohistochemical results showed that overexpression of MMP2 in thyroid tissues is associated with the occurrence of PTC and lymph node metastasis (LNM) [19]. Serum MMP-2 levels were found to be higher in PTC patients than in those with benign thyroid nodules (BTNs) or healthy individuals [20]. The serum MMP-2 level was also found to be an independent risk factor for poor prognosis among PTC patients after radiofrequency ablation, and its level was found to be decreased after treatment [21]. However, whether serum MMP-2 is a suitable biomarker for the prognosis of PTC after total or partial thyroidectomy has not been elucidated.

In this study, preoperative serum MMP-2 levels among PTC patients before total or partial thyroidectomy were examined, and their correlations with clinicopathological characteristics were assessed. Specifically, the diagnostic capacity of preoperative serum MMP-2 for distinguishing PTC from a BTN as well as its predictive performance for PTC prognosis were carefully evaluated.

## Materials and methods

### Study population

This study retrospectively reviewed the data of consecutive male patients with primary PTC treated at the First Hospital of Jilin University from July 2012 to August 2013. Age-matched, male subjects with BTNs as well as healthy individuals without any thyroid disease or nodules were also consecutively enrolled as controls from the Physical Examination Department. Healthy controls were volunteers who endowed the blood sample when they undertook blood test from July 2012 to September 2012. The diagnoses of PTC and BTN were made based on radiological findings from ultrasound, computed tomography scanning, and/or magnetic resonance imaging as well as histopathological results from fine-needle aspiration biopsy or thyroid tissue resection during/after thyroidectomy. The exclusion criteria were: 1) secondary PTC; 2) any anti-cancer therapy (radiation therapy, chemotherapy, thyroidectomy, etc.) prior to admission; 2) severe comorbidities or organ disorders, such as heart failure or malignant hematologic disease; and 3) insufficient data or records. This study was approved by the Ethics Committee of the First Hospital of Jilin University, and all participants gave their informed consent to participate.

The demographic and clinicopathological data of each PTC patient were collected. Age at the time of diagnosis was recorded. Body mass index (BMI) was calculated as weight in kilograms divided by the square of height in meters (kg/m^2^). All patients were classified at the time of diagnosis according to the American Joint Committee on Cancer tumor-node-metastasis (TNM) staging system (7^th^ edition) [22]. Early-stage disease is denoted as TNM stage I/II, and advanced-stage as stage III/IV.

### Thyroid surgery and follow-up

Total thyroidectomy with bilateral central-compartment neck dissection or partial thyroidectomy with unilateral central-compartment neck dissection was routinely performed in all patients with PTC. Lateral or modified neck dissection was conducted when metastatic cervical lymph nodes were suspected based on definitive clinical and/or imaging evidence. Central and lateral lymph node metastasis were diagnosed based on pathological findings. All surgical procedures were performed by the same team of surgeons.

Patients were followed up postoperatively every 3 months until August 2017. Serum MMP-2 level was assayed again three months after thyroidectomy. Radiological means (neck ultrasound, lung computed tomography scanning, or chest X-ray) and serum thyroglobulin (Tg) and thyroglobulin antibody (TgAb) levels were used to monitor postoperative PTC recurrence. A recurrence event was defined as structural evidence of disease identified after a period of no evidence of disease. Structurally persistent disease was defined as the occurrence of locoregional or distant metastasis regardless of Tg level [22]. The survival time without structurally persistent disease/recurrent disease (SPRD) was calculated as the interval between the initial treatment date and structurally persistent disease, recurrent disease, or the last follow-up.

### Determination of serum MMP-2 concentration

Preoperative fasting blood samples were collected from patients with PTC or BTNs at the time of diagnosis as well as healthy controls (HCs) at physical examination. Also, postoperative serum samples were collected from the PTC patients 3 months after initial thyroidectomy. Serum samples were obtained by centrifuging at 3000 × g for 10 min after 30 min of clotting time at room temperature. The serum samples were stored frozen at -70°C until analysis.

MMP-2 levels were determined in duplicate using a human MMP-2 enzyme-linked immunoassay kit (Boster Biological Technology, Wuhan, China) according to the manufacturer’s instructions [20]. Monoclonal antibody from mouse was used as the coating antibody, and polyclonal antibody from goat as the detection antibody. The limit of detection for the serum MMP-2 level was 10 pg/mL. The intra-assay coefficient of variation (CV) was <7%, and the inter-assay CV was 8%.

### Statistical analysis

SPSS software (version 18.0) was used for statistical analysis. Continuous data of normal and non-normal distribution are presented as mean ± standard deviation (SD) and median [interquartile range (IQR)], respectively. Categorical data are expressed as the number of patients and percentages. Continuous data were compared using an independent t test and analysis of variance (ANOVA) when the data were normally distributed and using nonparametric test when they were not. Proportions were compared using chi-squared test or nonparametric test.

Receiver operating characteristic (ROC) curves were constructed to determine the diagnostic power of serum MMP-2 for prediction of clinical outcomes. The sensitivity against the false-positive rate (1 –specificity) was plotted. Optimal cutoff values were defined for serum MMP-2 according to the Youden index [23]. The cumulative survival rates were calculated in a survival curve using the Kaplan–Meier method and compared using the log-rank test. Risk factors for predicting SPRD were identified by univariate and multivariate Cox proportional hazard analysis. Missing data from patients with PTC during follow-up were considered as censored. All statistical tests were two-sided, and statistical significance was determined as *p*<0.05.

## Results

### Characteristics of study population

The PTC, BTN and HC groups included 193, 53 and 69 subjects, respectively. There was no significant difference in age among the BTN group (44.81±12.70 years), HC group (40.93±12.94 years) and PTC group (42.39±10.43 years; *p*≥0.005). The clinicopathological characteristics of the participants are shown in Table 1.

**Table 1:** Clinicopathological characteristics of the study groups, n (%).

Characteristics	PTC (n = 193)	BTN (n = 53)	HC (n = 69)
Age			
<45 years	118 (61.1)	21 (39.6)	39 (56.5)
≥45 years	75 (38.9)	32 (60.4)	30 (43.5)
Tumor size			
≤1 cm	116 (60.1)		
>1 cm	77 (39.9)		
Capsule invasion			
No	67 (34.7)		
Yes	126 (65.3)		
Multifocality			
Unifocal	89 (46.1)		
Multifocal	104 (53.9)		
Nodal status			
N0	75 (38.9)		
N1a	109 (56.5)		
N1b	52 (26.9)		
Extrathyroidal invasion			
Negative	153 (79.3)		
Microscopic	30 (15.5)		
Macroscopic	10 (5.2)		
Vascular invasion			
No	176 (91.2)		
Yes	17 (8.8)		
Distant metastasis			
No	183 (94.8)		
Yes	10 (5.2)		
TNM stage			
I+II	149 (77.2)		
III+IV	44 (22.8)		

PTC, papillary thyroid carcinoma; BTN, benign thyroid nodule; HC, healthy control; TNM, tumor-node-metastasis.

### Comparison of preoperative serum MMP-2 concentrations among three groups

**Fig 1:**
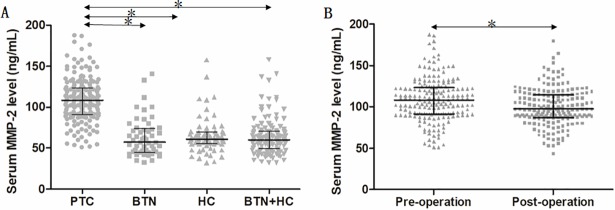
Serum MMP-2 concentrations of subjects. A Preoperative serum MMP-2 concentration in patients with PTC or BTH and in HCs. B preoperative versus postoperative serum MMP-2 concentration in the same patients with PTC. PTC, papillary thyroid carcinoma; BTN, benign thyroid nodule; HC, healthy control. The middle black horizontal lines indicate the median value of measurements, and the error bars indicate interquartile ranges. *p<0.001.

The median concentrations of preoperative serum MMP-2 were 57.15 ng/ml (IQR 44.69‒73.78 ng/ml) in the BTN group, 61.17 ng/ml (IQR 55.23‒69.25 ng/ml) in the HC group, and 60.28 ng/ml (IQR 49.01‒70.25 ng/ml) in the BTN+HC group (Fig 1A). There was no significant difference in the serum MMP-2 concentrations of the BTN and HC groups (*p* = 0.645). However, patients in the PTC group exhibited a significantly higher median serum MMP-2 concentration of 108.30 ng/ml (IQR 91.27‒123.58 ng/ml) than those in the BTN and HC groups (both *p*<0.001).

### Declination of postoperative serum MMP-2 in patients with PTC

Three months after thyroidectomy, abatement of serum MMP-2 in patients with PTC was observed in comparison of pre-operation (median 97.20 ng/ml, IQR 97.25‒114.71 *vs*. median 108.30 ng/ml, IQR 91.27‒123.58 ng/ml, p <0.001; Fig 1B).

### Role of MMP-2 in diagnosing PTC and predicting LNM

**Fig 2:**
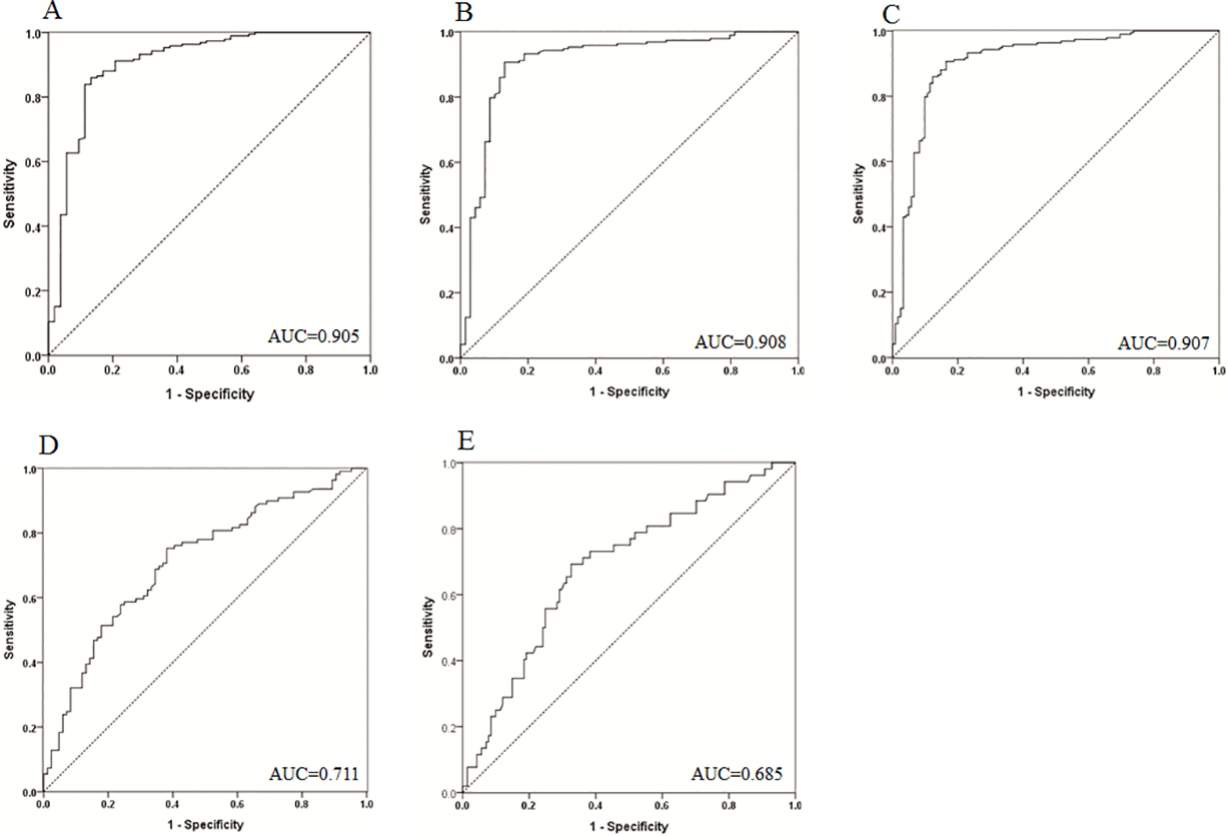
ROC curves for preoperative serum MMP-2 in the differential diagnosis for PTC (A‒C) and predicting LNM (D and E). A, Differential diagnosis of PTC from BTN; B, Differential diagnosis of PTC from HC; C, Differential diagnosis of PTC from BTN+HC; D, Prediction of CLNM. E, Prediction of LLNM. PTC, papillary thyroid carcinoma; BTN, benign thyroid nodule; HC, healthy control.

Based on the ROC curves constructed, preoperative serum MMP-2 at the cutoff value of 86.30 ng/ml had good performance for differentially diagnosing PTC versus BTN with an area under ROC curve (AUC) of 0.905. Similarly, at the cutoff value of 77.03 ng/ml and 70.16 ng/ml, serum MMP-2 had could differentiate PTC from HC (AUC 0.908) and BTN+HC (AUC 0.907), respectively, as shown in Table 2 and Fig 2A–2C.

**Table 2:** Performance of preoperative serum MMP-2 in differentially diagnosing PTC and predicting LNM.

	AUC (95% CI)	Optimum cut-off value (ng/ml)	Sensitivity (%)	Specificity (%)	PPV (%)	NPV (%)	LR+	LR-
PTC diagnosis								
PTC *vs*. BTN	0.905 (0.851–0.958)	86.30	86.0	86.8	96.0	63.0	6.515	0.161
PTC *vs*. HC	0.908 (0.861–0.956)	77.03	90.7	87.0	95.1	76.9	6.977	0.107
PTC *vs*. BTN+HC	0.907 (0.869–0.944)	70.16	93.3	73.8	84.9	87.4	3.561	0.091
LNM								
CLNM	0.711 (0.638–0.784)	101.55	75.2	61.9	71.9	65.8	1.974	0.407
LLNM	0.685 (0.602–0.769)	112.67	69.2	67.4	43.9	85.6	2.123	0.457

PTC, papillary thyroid carcinoma; BTN, benign thyroid nodule; HC, healthy control; LNM, lymph node metastasis; CLNM, central lymph node metastasis; LLNM, lateral lymph node metastasis; AUC, area under the ROC curve; CI, confidence interval; PPV, positive predictive value; NPV, negative predictive value; LR+, positive likelihood ratio; LR-, negative likelihood ratio.

Preoperative serum MMP-2 had a moderate predictive capacity for central LNM (CLNM) with an AUC of 0.711 and lateral LNM (LLNM) with an AUC of 0.685 (Table 2, Fig 2D and 2E).

### Association between preoperative serum MMP-2 concentration and clinicopathological characteristics

Patients with PTC were subdivided into low MMP-2 (<86.30 ng/ml) and high MMP-2 (≥86.30 ng/ml) groups, based on the cutoff value of serum MMP-2 at 86.30 ng/ml for differentiating PTC from BTN. Patients with high MMP-2 had greater risks of developing a larger tumor (size >1 cm, *p*<0.001), CLNM (*p* = 0.002), LLNM (*p* = 0.046), extrathyroidal invasion (*p* = 0.004), and advanced TNM stage (*p* = 0.011) than those with low MMP-2 (Table 3).

**Table 3:** Comparison of clinicopathological variables in PTC patients based on stratified preoperative serum MMP-2 concentration.

	Serum MMP-2 (ng/mL)		*p* value
	Low level (<86.30), (n = 27)	High level (≥86.30), (n = 166)	
Age			0.525
<45 years	18 (66.7)	100 (60.2)	
≥45 years	9 (33.)	66 (39.8)	
Tumor size			**<0.001**
≤1cm	25 (92.6)	91 (54.8)	
>1 cm	2 (7.4)	75 (45.2)	
Capsule invasion			0.478
No	11 (40.7)	56 (33.7)	
Yes	16 (59.3)	110 (66.3)	
Multifocality			0.819
No	13 (48.1)	76 (45.8)	
Yes	14 (51.9)	90 (54.2)	
CLNM			
No	19 (70.4)	65 (39.2)	**0.002**
Yes	8 (29.6)	101 (60.8)	
LLNM			**0.046**
No	24 (88.9)	117 (70.5)	
Yes	3 (11.1)	49 (29.5)	
Extrathyroidal invasion			**0.004**
No	27 (100.0)	126 (75.9)	
Yes	0 (0.0)	40 (24.1)	
Vascular invasion			0.169
No	27 (100.0)	149 (89.8)	
Yes	0 (0.0)	17 (10.2)	
Distant metastasis			0.352
No	27 (100.0)	155 (93.4)	
Yes	0 (0.00)	11 (6.6)	
TNM stage			**0.011**
I+II	26 (96.3)	123 (74.1)	
III+IV	1 (3.7)	43 (25.9)	

PTC, papillary thyroid carcinoma; CLNM, central lymph node metastasis; LLNM, lateral lymph node metastasis; TNM, tumor-node-metastasis.

*p*-values were determined by Chi-square test.

### Risk factors for LNM in male patients with PTC

Based on the optimum cutoff value of preoperative serum MMP-2 for predicting CLNM (101.55 ng/ml), PTC patients were stratified into two subsets (MMP-2 ≥101.55 ng/ml *vs*. <101.55 ng/ml). Multivariate logistic regression analysis demonstrated that older age (≥45 years, *p*<0.001), high MMP-2 concentration (*p* = 0.001), and advanced TNM stage (*p* = 0.001) were independent risk factors for developing CLNM (Table 4).

**Table 4:** Risk factors for LNM by multivariate logistic regression.

	CLNM		LLNM	
	OR (95% CI)	*p* value	OR (95% CI)	*p* value
Age (≥45 years *vs* < 45 years)	10.989 (2.886–41.887)	**0.000**	2.977 (0.452–5.174)	0.998
BMI (≥25 kg/m^2^ *vs*. <25 kg/m^2^)	1.160 (0.532–2.529)	0.710	2.210 (0.853–5.727)	0.103
Preoperative serum MMP-2 (≥101.55 ng/ml *vs*. <101.55 ng/ml	3.627 (1.665–7.901)	**0.001**		
Preoperative serum MMP-2 (≥112.67 ng/ml *vs*. <112.67 ng/ml)			2.318 (0.954–5.633)	0.063
Tumor size (>1 cm *vs*. ≤1 cm)	0.890 (0.365–2.171)	0.798	1.106 (0.405–3.021)	0.844
Capsule invasion (yes *vs*. no)	1.375 (0.626–3.021)	0.427	0.537 (0.223–1.294)	0.166
Multifocality (yes *vs*. no)	1.091 (0.524–2.273)	0.816	1.749 (0.782–3.913)	0.174
Extrathyroidal invasion (yes *vs*. no)	1.695 (0.537–5.347)	0.368	0.784 (0.248–2.480)	0.679
Vascular invasion (yes *vs*. no)	1.138 (0.237–5.472)	0.872	2.043 (0.553–7.540)	0.284
Distant metastasis (yes *vs*. no)	0.411 (0.081–2.080)	0.282	3.603 (0.701–18.510)	0.125
TNM stage (III+IV *vs*. I+II)	13.591 (2.896–63.790)	**0.001**	3.799 (0.873–10.258)	0.612

BMI, body mass index; TNM, tumor-node-metastasis; CLNM, central lymph node metastasis; LLNM, lateral lymph node metastasis.

Similarly, the PTC patients were stratified into those with MMP-2 ≥112.67 ng/ml versus <112.67 ng/ml based on the optimum cutoff value of preoperative serum MMP-2 for predicting LLNM. However, none of examined variables were found to be risk factors for developing LLNM.

### Significance of MMP-2 in predicting survival during the 4-year follow-up

PTC patients with SPRD had a significant higher median preoperative serum MMP-2 level (149.22 ng/ml, IQR 113.39‒170.96 ng/ml) than that in those without SPRD (104.55 ng/ml, IQR 91.24‒120.88 ng/ml; *p*<0.001).

**Fig 3:**
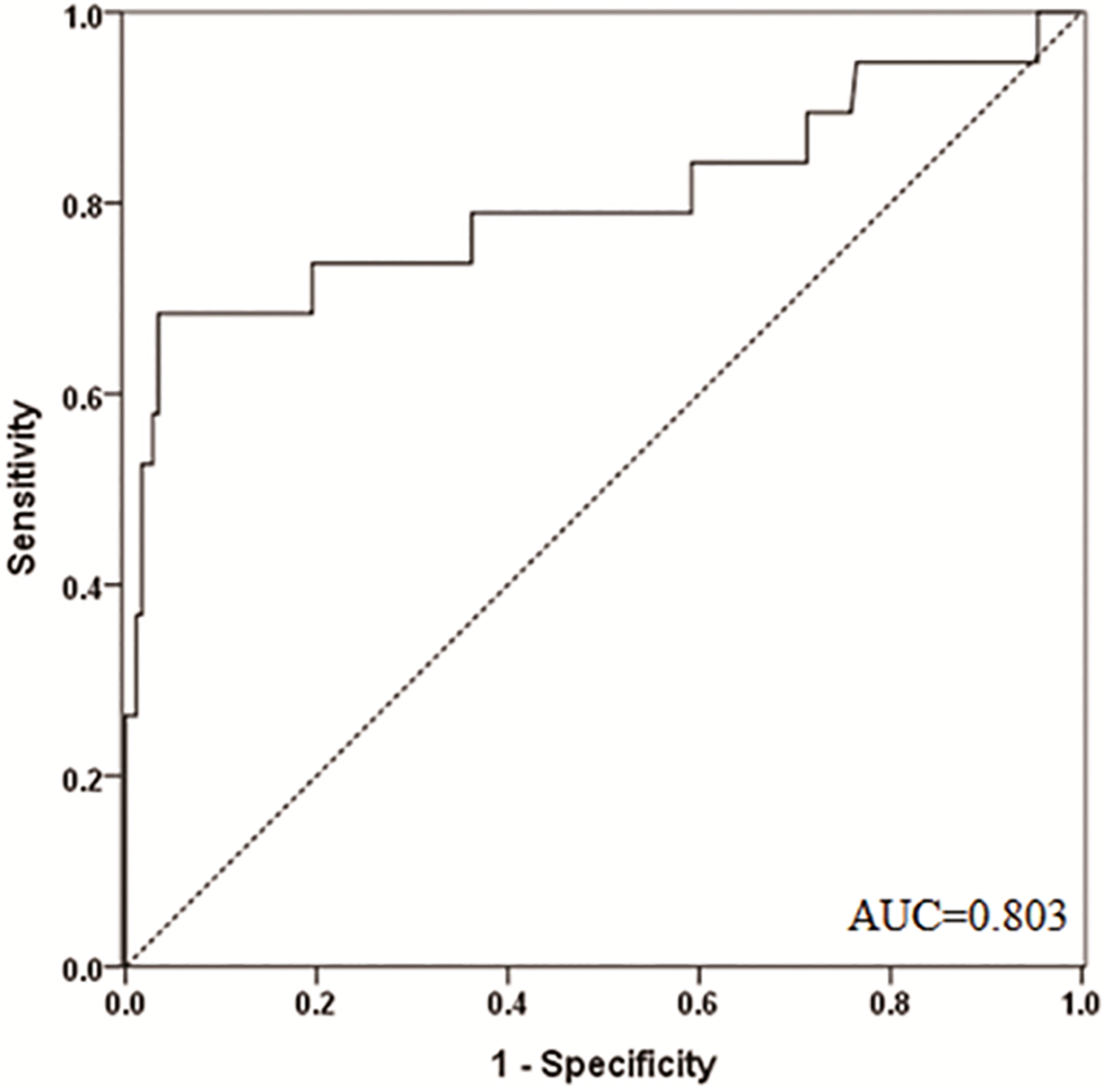
ROC curve for the ability of preoperative serum MMP-2 to predict SPRD.

Preoperative serum MMP-2 at the cutoff value of 144.04 ng/ml was able to predict survival without SPRD, with an AUC of 0.803 (95% CI 0.664‒0.942), a sensitivity of 68.4%, and a specificity of 97.0% (Fig 3).

Patients with older age (≥45 years, *p* = 0.001) were more likely to have SPRD than patients with younger age (<45 years). Patients tended to develop SPRD if they had greater tumor size (>1 cm, *p* = 0.001), clinicopathological features of invasiveness [capsule invasion (*p* = 0.005), CLNM (*p* = 0.010), extrathyroidal invasion (*p*<0.001), vascular invasion (*p*<0.001), distant metastasis (*p* = 0.012)], and advanced TNM stage (*p*<0.001), Table 5.

**Table 5:** Comparison of clinicopathological variables in PTC patients with or without SPRD, n (%).

Features	Without SPRD (n = 174)	SPRD (n = 19)	*p* value
Age			**0.001**
<45 years	113 (64.9)	5 (26.3)	
≥45 years	61 (35.1)	14 (73.7)	
Tumor size			**0.002**
≤1 cm	111 (63.8)	5 (26.3)	
>1 cm	63 (36.2)	14 (73.7)	
Capsule invasion			**0.005**
No	66 (37.9)	1 (5.3)	
Yes	108 (62.1)	18 (94.7)	
Multifocality			0.181
No	83 (47.7)	6 (31.6)	
Yes	91 (52.3)	13 (68.4)	
CLNM			**0.010**
No	81 (46.6)	3 (15.8)	
Yes	93 (53.4)	16 (84.2)	
LLNM			0.306
No	129 (74.1)	12 (63.2)	
Yes	45 (25.9)	7 (36.8)	
Extrathyroidal invasion			**<0.001**
No	145 (83.3)	8 (42.1)	
Yes	29 (16.7)	11 (57.9)	
Vascular invasion			**<0.001**
No	165 (94.8)	11 (57.9)	
Yes	9 (5.2)	8 (42.1)	
Distant metastasis			**0.012**
No	167 (96.0)	15 (78.9)	
Yes	7 (4.0)	4 (21.1)	
TNM stage			**<0.001**
I+II	145 (83.3)	4 (21.1)	
III+IV	29 (16.7)	15 (78.9)	

PTC, papillary thyroid carcinoma; CLNM, central lymph node metastasis; LLNM, lateral lymph node metastasis; TNM, tumor-node-metastasis.

*p* values were determined by Chi-square.

**Fig 4:**
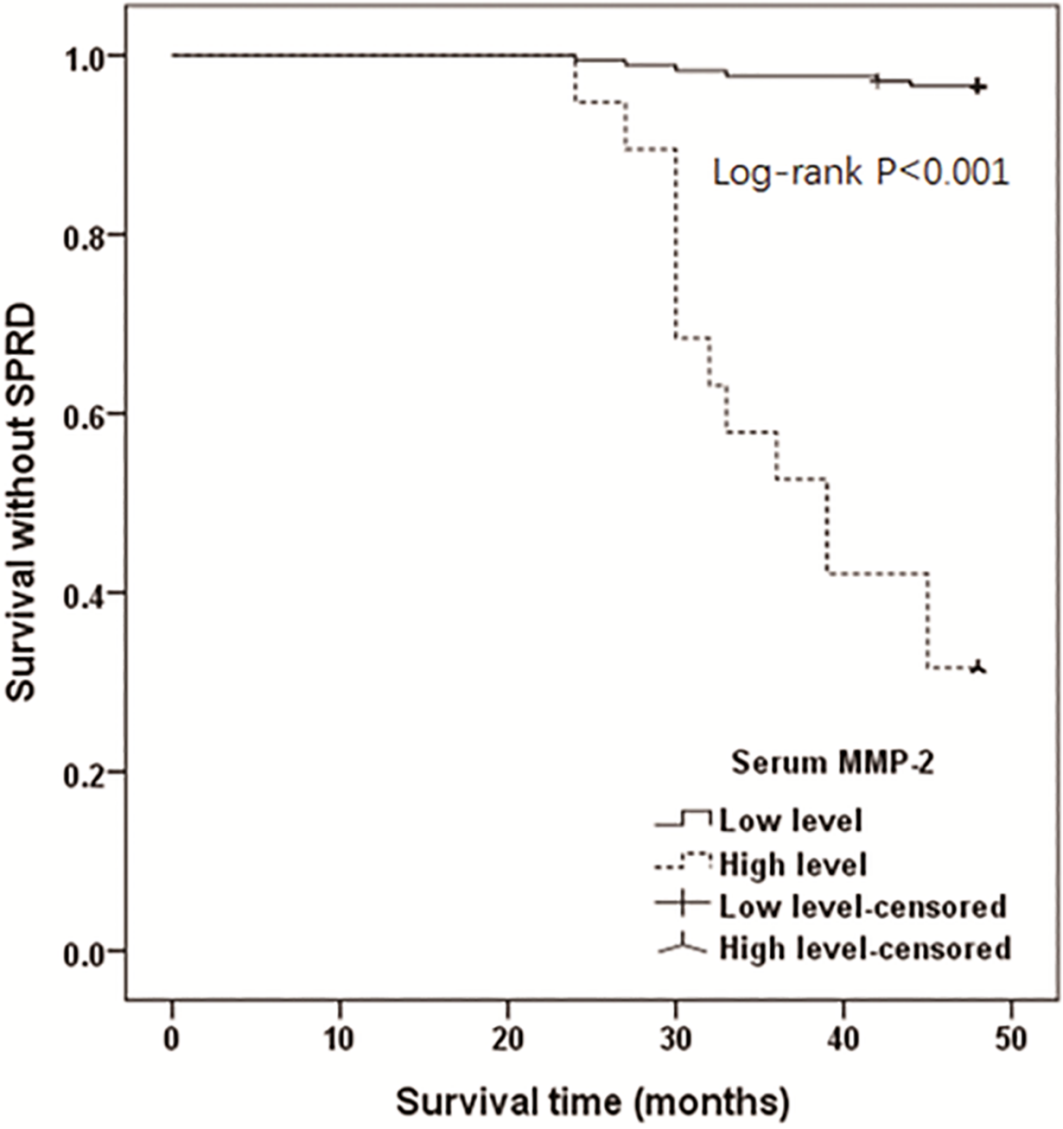
Survival without SPRD in PTC patients stratified by preoperative serum MMP-2 level (low level <144.04 ng/ml; high level ≥144.04 ng/ml).

In addition, the mean survival time without SPRD in all PTC patients was 46.56±4.77 months, and the 4-year rate of survival without SPRD in all PTC patients was 90.16%. These patients were subdivided into a high serum MMP-2 level group (≥144.04 ng/ml) and a low serum MMP-2 level group (<144.04 ng/ml). The survival time without SPRD in patients with high preoperative serum MMP-2 was significantly shorter than that in patients with low preoperative serum MMP-2 (38.32±8.62 months vs. 47.46±3.06 months, p<0.001). In addition, the 4-year rate of survival without SPRD in patients with high preoperative serum MMP-2 was significantly lower than that in patients with low preoperative serum MMP-2 (31.6% *vs*. 96.6%, *p*<0.001). Patients with a high serum MMP-2 level (≥144.04 ng/ ml) had a worse clinical outcome than those with a low serum MMP-2 level (<144.04 ng/ml; *p*<0.001 by log-rank test, Fig 4).

### Prognostic factors for SPRD in male patients with PTC

Univariate Cox regression model demonstrated that age ≥45 years (*p* = 0.003), tumor size >1 cm (*p* = 0.005), capsule invasion (*p* = 0.023), CLNM (*p* = 0.021), extrathyroidal invasion (*p*<0.001), vascular invasion (*p*<0.001), distant metastasis (*p* = 0.003), advanced TNM stage (*p*<0.001), and high preoperative serum MMP-2 (≥144.04 ng/ml, *p*<0.001) were risk factors for SPRD. These variables were then introduced into the multivariate Cox regression. The results showed that only advanced TNM stage (odds ratio [OR] 42.255, 95% confidence interval [CI] 2.324‒768.286, *p* = 0.011) and high preoperative serum MMP-2 level (OR 15.231, 95% CI 4.101‒56.568, *p*<0.001) were independent risk factors of SPRD (Table 6).

**Table 6:** Prognostic factors for SPRD in PTC patients by Cox regression model.

	Univariate analysis		Multivariate analysis	
	OR (95% CI)	*p* value	OR (95% CI)	*p* value
Age (≥45 years *vs*. <45 years)	4.734 (1.705–13.45)	**0.003**	0.118 (0.007–1.938)	0.135
BMI (≥25 kg/m^2^ *vs*. < 25 kg/m^2^)	0.920 (0.362–2.337)	0.861		
Tumor size (>1 cm *vs*. ≤1 cm)	4.394 (1.582–12.202)	**0.005**	0.814 (0.185–3.573)	0.785
Capsule invasion (yes *vs*. no)	10.278 (1.372–77.000)	**0.023**	7.969 (0.957–66.381)	0.055
Multifocality (yes *vs*. no)	1.892 (0.719–4.978)	0.267		
CLNM (yes *vs*. no)	4.273 (1.245–14.668)	**0.021**	0.894 (0.173–4.607)	0.893
LLNM (yes *vs*. no)	1.619 (0.637–4.112)	0.311		
Extrathyroidal invasion (yes *vs*. no)	5.655 (2.273–14.069)	**<0.001**	1.053 (0.265–4.186)	0.941
Vascular invasion (yes *vs*. no)	8.452 (3.395–21.046)	**<0.001**	2.177 (0.585–8.101)	0.246
Distant metastasis (yes *vs*. no)	5.209 (1.726–15.721)	**0.003**	0.258 (0.049–1352)	0.109
TNM stage (III+IV *vs*. I+II)	15.177 (5.030–45.791)	**<0.001**	42.255 (2.324–768.286)	**0.011**
Preoperative serum MMP-2 (≥144.04 ng/ml *vs*. <144.04 ng/ml)	29.064 (10.932–77.268)	**<0.001**	15.231 (4.101–56.568)	**<0.001**

PTC, papillary thyroid carcinoma; BMI, body mass index; CLNM, central lymph node metastasis; LLNM, lateral lymph node metastasis; TNM, tumor-node-metastasis.

## Discussion

In this study, patients with PTC exhibited a higher level of preoperative serum MMP-2 (median 108.30 ng/ml *vs*. 57.15 ng/ml for those with a BTN), especially in those with SPRD (149.22 ng/ml vs.104.55 ng/ml for those without SPRD). MMP-2 concentration abated in patients with PTC at 3 months after thyroidectomy. Patients with a lower preoperative serum MMP-2 level (<144.04 ng/ml) had a better clinical outcome than those with a higher preoperative serum MMP-2 level (≥144.04 ng/ml). Preoperative serum MMP-2 concentration was an independent risk factor for both CLNM and SPRD, indicating that MMP-2 is involved in the pathogenesis and progression of PTC. Importantly, the cutoff values of preoperative serum MMP-2 were identified for diagnosing PTC (86.30 ng/ml), predicting CLNM (101.55 ng/ml), and predicting SPRD (144.04 ng/ml). To our knowledge, this study for the first time determined the performance of preoperative serum MMP-2 in predicting SPRD in male patients with PTC after total or partial thyroidectomy. Thus, determination of preoperative serum MMP-2 provides a novel and potentially valuable tool for diagnosis and prognosis of PTC in clinical practice.

MMP-2, as a type of zinc-dependent endopeptidase, functions to digest gelatin and multiple types of collagens and has been implicated in the development of various tumors [24,25]. MMP-2 has been reported to be related to tumorigenesis, tumor invasiveness [10], and metastasis [14,26]. A meta-analysis demonstrated that MMP-2 expression was higher in ovarian cancer tissue than that in benign or normal ovarian tissue [27]. Overexpression of MMP-2 in thyroid tissue may be correlated with the occurrence of PTC [19]. Some studies have explored the role of serum/plasma MMP-2 in tumorigenesis. A study demonstrated an increase in plasma MMP-2 in colorectal cancer over benign colonic pathology [28]. The serum MMP-2 level was also found to be associated with the development of gastric cancer [29]. In this study, serum MMP-2 concentration was elevated in the PTC group as compared with the controls. This finding was consistent with a previous study, which confirmed that patients with PTC had a higher serum MMP-2 level than controls [20,30]. Moreover, serum MMP-2 level decreased after thyroidectomy, in accordance with previous study reported [21].We found that preoperative serum MMP-2 at the cutoff value of 86.30 ng/ml had good performance for the diagnosis of PTC, with a sensitivity of 86.0% and a specificity of 86.8%. In the study performed by Kumara et al., the preoperative plasma MMP-2 was reported to discriminate colorectal cancer from benign tissue with a sensitivity of 55% and a specificity of 80% [28]. The discrepancy in sensitivity/specificity may have resulted from the different tumor types and samples collected. In fact, serum MMP-2 levels differ in plasma and serum, as MMP-2 is expressed at a higher concentration in serum than in plasma [31].

In this study, PTC patients with a high preoperative serum MMP-2 (≥86.30 ng/ml) were more likely to have a larger tumor size, presence of CLNM, LLNM, extrathyroidal invasion, and advanced TNM stage. On the other hand, preoperative serum MMP-2 (≥101.55 ng/ml) was positively correlated with CLNM. These data indicate that serum MMP-2 is tightly associated with invasive characteristics of PTC. Similar results have been obtained in previous studies. Pancreatic carcinoma patients with lymph node involvement and advanced TNM stage exhibit a higher MMP-2 level than patients without [32]. In PTC, MMP-2 was found to be associated with LNM by immunohistochemical analysis [33]. Wu et al found that the level of MMP-2 in PTC tissue is positively associated with LLNM [34]. MMP-2 is capable of disrupting the basement membrane by degrading type IV collagen and extracellular matrix. In addition, MMP-2 is involved in angiogenesis, which promotes the progression, invasion and metastasis of tumors [28,35]. These factors may partially explain the role of MMP-2 in PTC invasiveness.

Our findings demonstrate that a high preoperative serum level of MMP-2 (≥144.04 ng/ml) performed well in predicting SPRD in male PTC patients, and MMP-2 was an independent risk factor for SPRD. Inconsistent with our results, Tabouret et al. found that a high level of serum MMP-2 was associated with better disease-free survival and overall survival in patients with HER2(+) inflammatory breast cancer who were treated with bevacizumab and trastuzumab, as compared with those with low serum MMP-2 [17]. A meta-analysis including 4944 breast cancer patients revealed that overexpression of MMP-2 was not related to shorter overall survival [36]. These discrepancies may be due to differences in the types of carcinomas, chemotherapy, and detection assays among these studies. However, our results were in line with the results of He et al. [21], who found that a high serum MMP-2 concentration correlated with worse prognosis of PTC after radiofrequency ablation. The correlation between serum MMP-2 and clinical outcome is possibly due to the link between the MMP-2 level and tumor burden, such as lymph node involvement and advanced TNM stage.

There are several strengths in the present study. First, to the best of our knowledge, this is the first study to analyze the correlation between preoperative serum MMP-2 and PTC prognosis after thyroidectomy. Second, only male patients were included in the study to minimize the selection bias caused by gender. This study also has some limitations. First, this is a retrospective study with a limited sample size that was performed in a single center, which makes the conclusion less reliable. Second, it has been reported that physical activity may modulate serum MMP-2 concentration [37]. In this study, unmeasured factors, such as physical activity, that could have possibly influenced the results were not taken into account when serum MMP-2 concentrations were determined. In addition, there was no control serum biomarker due to a lack of well-known serological biomarkers for PTC diagnosis in clinical practice.

In conclusion, preoperative serum MMP-2 may serve as a potential biomarker for the diagnosis of PTC and prediction of CLNM and SPRD in these patients. Further investigations are necessary to advance our understanding of the mechanism by which MMP-2 promotes tumorigenesis and progression of PTC.
